# Right ventricular injury in critically ill patients with COVID-19: a descriptive study with standardized echocardiographic follow-up

**DOI:** 10.1186/s13613-024-01248-8

**Published:** 2024-01-23

**Authors:** Mathieu Jozwiak, Claire Dupuis, Pierre Denormandie, Didac Aurenche Mateu, Jean Louchet, Nathan Heme, Jean-Paul Mira, Denis Doyen, Jean Dellamonica

**Affiliations:** 1 Service de Médecine Intensive Réanimation, Hôpitaux Universitaires Paris Centre, Hôpital Cochin, Assistance Publique–Hôpitaux de Paris, 27 Rue du Faubourg Saint Jacques, 75014 Paris, France; 2 Université Paris Cité, Paris, France; 3 UR2CA-Unité de Recherche Clinique Côte d’Azur, Université Côte d’Azur, Nice, France; 4 Service de Médecine Intensive Réanimation, Centre Hospitalier Universitaire de Clermont-Ferrand, Hôpital Gabriel Montpied, 58 Rue Montalembert, 63000 Clermont-Ferrand, France; 5 IAME Université Paris Cité, U 1137, 75018 Paris, France; 6 Service de Médecine Intensive Réanimation, Centre Hospitalier Universitaire de Nice, Hôpital L’Archet 1, 151 Rue Saint Antoine de Ginestière, 06200 Nice, France

**Keywords:** Acute respiratory distress syndrome, COVID-19, Cardiac injury, Right ventricle, Prognosis

## Abstract

**Purpose:**

Patients with COVID-19 admitted to intensive care unit (ICU) may have right ventricular (RV) injury. The main goal of this study was to investigate the incidence of RV injury and to describe the patient trajectories in terms of RV injury during ICU stay.

**Methods:**

Prospective and bicentric study with standardized transthoracic echocardiographic (TTE) follow-up during ICU stay with a maximum follow-up of 28 days. The different patterns of RV injury were isolated RV dilation, RV dysfunction (tricuspid annular plane systolic excursion < 17 mm and/or systolic tricuspid annular velocity < 9.5 cm/s and/or RV fractional area change < 35%) without RV dilation, RV dysfunction with RV dilation and acute cor pulmonale (ACP, RV dilatation with paradoxical septal motion). The different RV injury patterns were described and their association with Day-28 mortality was investigated.

**Results:**

Of 118 patients with complete echocardiographic follow-up who underwent 393 TTE examinations during ICU stay, 73(62%) had at least one RV injury pattern during one or several TTE examinations: 29(40%) had isolated RV dilation, 39(53%) had RV dysfunction without RV dilation, 10(14%) had RV dysfunction with RV dilation and 2(3%) had ACP. Patients with RV injury were more likely to have cardiovascular risk factors, to be intubated and to receive norepinephrine and had a higher Day-28 mortality rate (27 vs. 7%, *p* < 0.01). RV injury was isolated in 82% of cases, combined with left ventricular systolic dysfunction in 18% of cases and 10% of patients with RV injury experienced several patterns of RV injury during ICU stay. The number of patients with de novo RV injury decreased over time, no patient developed de novo RV injury after Day-14 regardless of the RV injury pattern and 20(31%) patients without RV injury on ICU admission developed RV injury during ICU stay. Only the combination of RV dysfunction with RV dilation or ACP (aHR = 3.18 95% CI(1.16–8.74), *p* = 0.03) was associated with Day-28 mortality.

**Conclusion:**

RV injury was frequent in COVID-19 patients, occurred within the first two weeks after ICU admission and was most often isolated. Only the combination of RV dysfunction with RV dilation or ACP could potentially be associated with Day-28 mortality.

*Clinical trial registration* NCT04335162.

**Supplementary Information:**

The online version contains supplementary material available at 10.1186/s13613-024-01248-8.

## Background

Up to 77% of COVID-19 patients admitted to intensive care unit (ICU) had RV injury during ICU stay and several patterns of RV injury have been described, from isolated RV dilation to acute cor pulmonale (ACP) [1–12]. RV injury is likely to be related to increased RV afterload due to acute respiratory distress syndrome, mechanical ventilation, respiratory worsening, and/or pulmonary thromboembolism [8, 13], but probably also, to a lesser extent, to impaired intrinsic contractility as seen in some viral myocarditis [14]. Regardless of its mechanisms, it has been suggested that RV injury in critically ill COVID-19 patients may be associated [4, 6, 8–12] or not [5] to mortality. However, most of these studies were retrospective [4–6, 11, 12], included a single echocardiographic examination performed early in ICU stay [4, 5, 9, 10] or included several echocardiographic examinations performed at different times between patients without standardized echocardiographic follow-up during ICU stay [6, 8, 11, 12].

In this prospective study, we performed systematic and content-standardized echocardiographic follow-up in critically ill COVID-19 patients during ICU stay at predetermined times not clinically driven, with respiratory characteristics available at the time of each echocardiographic assessment. The main goal of this study was to investigate the incidence of RV injury and its severity over time, as well as to describe the different patterns of RV injury during ICU stay.

## Methods and patients

This prospective and observational study was conducted in two intensive care units (ICUs) of French University hospitals and was approved by the Ethics committee of Nice hospital (number R04-022 3313140420) and complies with the current revision of the Declaration of Helsinki. Informed consent was waived but all patients or next-of-kin were informed about the study. The study complied with the Strengthening the Reporting of Observational Studies in Epidemiology (STROBE) [15] and PRICES [16] statement guidelines.

### Patients

We included during the first two pandemic waves (March 2020 to March 2021) all consecutive patients who were 18 years of age or older and admitted to ICU for severe COVID-19 pneumonia. All patients had a positive result on a real-time reverse transcriptase-polymerase chain reaction assay for SARS-CoV-2 from nasal swabs. Exclusion criteria were pregnancy, patients with non-diagnostic echocardiographic windows, which was defined as the inability to accurately align the Doppler beam for reliable Doppler measurements and/or delineate the endocardium to measure the left and right ventricular end-diastolic area (LVEDA and RVEDA), as well as patients with a decision to withdraw life-sustaining therapy.

### Transthoracic echocardiographic measurements

A transthoracic echocardiography (TTE) examination was performed in all patients on ICU admission (Day-0), on Day-3, on Day-7 and then weekly until ICU discharge or death, with a maximum follow-up of 28 days. In each ICU, the TTE examinations were performed by the same experienced board-certified operators using a Philips CX 50 (Philips Healthcare, DA Best, The Netherlands) or a Vivid E9 (GE Healthcare, Horten, Norway) and TTE variables were measured at end-expiration following the current recommendations [17]. TTE measurements were averaged on three consecutive measurements in patients with sinus rhythm and five consecutive measurements in patients with atrial fibrillation [18]. All contours were hand-drawn. Besides echocardiographic parameters of RV function, usual echocardiographic parameters of LV systolic and diastolic function were also collected. The RV-pulmonary arterial coupling was assessed by calculating the ratio of tricuspid annular plane systolic excursion (TAPSE) to systolic pulmonary artery pressure and the ratio of RV fractional area change to systolic pulmonary artery pressure [19–21].

### Definition of RV injury

**Fig. 1:**
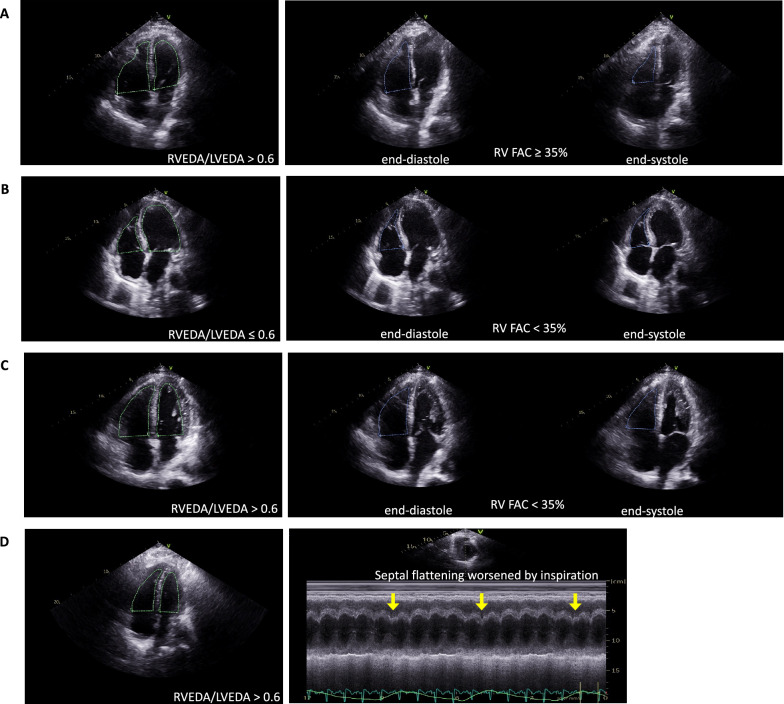
Echocardiographic views illustrating the four different right ventricular (RV) injury patterns. **A** isolated RV dilation (apical 4-chamber view). **B** RV dysfunction without RV dilation (apical 4-chamber view). **C** RV dysfunction with RV dilation (apical 4-chamber view). **D** acute cor pulmonale (apical 4-chamber view) with flattening of the interventricular septum worsened by inspiration (yellow arrows, short-axis view in M-Mode). RVEDA: right ventricular end-diastolic area; LVEDA: left ventricular end-diastolic area; RV-FAC: right ventricular fractional area change

Four different patterns of RV injury were defined: isolated RV dilation, RV dysfunction without RV dilation, RV dysfunction with RV dilation and ACP (Fig. 1 and Additional file 2: Fig. S1). RV dilation was defined by a RVEDA/LVEDA ratio > 0.6 without paradoxical septal motion [4, 8]. RV dysfunction was defined by at least one of the following criteria: TAPSE < 17 mm and/or systolic tricuspid annular velocity < 9.5 cm/s and/or RV fractional area change < 35% [17]. ACP was defined by a RVEDA/LVEDA ratio > 0.6 with a paradoxical septal motion [22].

### Data collection and endpoints

Patient characteristics, clinical and biological variables as well as therapeutics were collected on ICU admission and at the time of TTE examination. Clinical outcomes were collected during ICU stay with a maximum follow-up at ICU discharge or until death if earlier.

The primary endpoint of the study was the proportion of patients with RV injury during ICU stay. Secondary endpoints were the time of occurrence of RV injury, the proportion of patients with the different RV injury patterns during ICU stay, the Day-28, Day-90 and ICU mortality rates, the duration of mechanical ventilation, the length of ICU stay and the risk factors for Day-28 mortality rate.

### Statistical analyses

Continuous variables were expressed as median (interquartile range) and categorical variables as number (percentages). Comparisons were performed for continuous and categorical variables using Pearson’s Chi‐ Squared test or exact Fisher’s test, and Wilcoxon’s rank sum test, as appropriate. The interobserver variability was assessed in 12 randomly selected patients using intraclass correlation coefficients for LV ejection fraction, LVEDA, TAPSE, systolic tricuspid annular velocity, RVEDA, RV fractional area change and RVEDA/LVEDA ratio.

First, we assessed the impact of RV injury on Day-28 mortality rate using a time‐dependent Cox model. RV injury and all variables at the time of TTE examination (intubation, positive end-expiratory pressure (PEEP) level, driving pressure, partial arterial pressure of oxygen over inspired oxygen fraction ratio, partial arterial pressure of carbon dioxide (PaCO_2_), norepinephrine administration and norepinephrine dosage) were considered as time-dependent covariates. All variables with a *p* value < 0.2 at univariate analyses were entered in the different multivariable models. Then, a backward selection based on the Bayesian information criterion was achieved to select the final model. The proportionality of hazard risks (HR) for the covariates was assessed using the marginal residuals. Collinearity between covariates was checked. Covariates were dichotomized if necessary, based on their median values on ICU admission or usual cut-off values. RV injury was forced in all models. Second, a mixed effect logistic regression model was achieved to take into account for individual changes. All models were stratified by centre. The incidence of death up to Day-28 according to the most severe RV injury pattern during ICU stay was illustrated using a cumulative incidence curve. Missing variables were handled through multiple imputation with only one dataset. All tests were two-sided and a *p*-value < 0.05 was considered statistically significant. All statistical analyses were performed using the SAS software, Version 9.4 (SAS Institute, Cary, NC, USA), and R (version 3.6.3, R foundation for Statistical Computing Vienna, Austria).

## Results

### Study population

Out of the 188 patients admitted to the two ICUs during the study period, 19(10%) were excluded due to non-diagnostic echocardiographic windows, 40(21%) due to incomplete follow-up and 11(6%) due to a decision to withdraw life-sustaining therapy. Overall, 118 patients were included and 393 TTE examinations were performed with 3(2–4) TTE examinations per patient. The median age was 65(60–73) years, 89(75%) patients had cardiovascular risk factors, 85(72%) were mechanically ventilated and 68(58%) received norepinephrineduring ICU stay (Table 1).

**Table 1:** Patient characteristics and management according to the presence of RV injury during ICU stay

	No RV injury (*n* = 45)	RV injury (*n* = 73)	*p*-value
Clinical characteristics			
Age (years)	64 (59–70)	67 (61–74)	0.20
Gender (male), *n* (%)	33 (73)	51 (70)	0.69
SAPS-2 score	38 (29–51)	45 (34–67)	0.05
SOFA score on ICU admission	6 (3–9)	8 (4–11)	0.12
Body mass index (kg/m^2^)	28 (26–30)	28 (26–31)	0.90
Obesity, *n* (%)	13 (29)	27 (37)	0.37
Arterial hypertension, *n* (%)	18 (40)	40 (55)	0.12
Diabete mellitus, *n* (%)	10 (22)	25 (34)	0.16
Dyslipidemia, *n* (%)	7 (16)	24 (33)	0.04
Smokers, *n* (%)	8 (18)	9 (12)	0.41
Coronary artery disease, *n* (%)	4 (9)	7 (10)	0.90
Stroke, *n* (%)	1 (2)	5 (7)	0.27
Chronic heart failure, *n* (%)	2 (4)	2 (3)	0.62
Chronic respiratory disease, *n* (%)	0 (0)	5 (7)	0.07
Chronic kidney disease, *n* (%)	5 (11)	15 (21)	0.18
Immunosuppression, *n* (%)	5 (11)	19 (26)	0.05
Renin-Angiotensin System Blockers, *n* (%)	15 (33)	31 (43)	0.32
Delay from onset of symptoms to ICU admission (days)	8 (7–11)	9 (6–12)	0.98
Oxygenation variables at the time of TTE examination on ICU admission			
pH	7.50 (7.40- 7.50)	7.40 (7.40–7.50)	0.14
FiO_2_ (%)	0.7 (0.5–0.9)	0.6 (0.5–0.8)	0.48
PaO_2_/FiO_2_	121 (80–190)	112 (79–165)	0.50
PaCO_2_ (mmHg)	35 (32–40)	39 (33–43)	0.05
Blood lactate level (mmol/L)	1.0 (0.8–1.3)	1.2 (1.0–1.5)	0.03
Biological variables on ICU admission			
Leukocytes (G/L)	8.5 (7.0–11.0)	8.4 (6.7–11.2)	0.95
Neutrophils (G/L)	7.4 (6.1–9.9)	7.3 (5.6–10.3)	0.93
Platelet count (G/L)	228 (186–287)	232 (180–292)	1.00
Lymphocytes (G/L)	0.7 (0.5–0.9)	0.7 (0.5–0.9)	0.87
Fibrinogen (g/L)	5.7 (4.8–7.3)	6.4 (5.4–7.7)	0.14
D-Dimers (µg/L)	1099 (679–2255)	1206 (709–2256)	0.69
Protein C reactive (mg/L)	122 (71–268)	136 (91–207)	0.93
Procalcitonin (ng/L)	0.30 (0.20–0.80)	0.30 (0.10–0.80)	0.80
Ferritin (ng/mL)	1066 (575–1949)	1096 (781–2751)	0.38
Interleukin-6 (pg/mL)	70 (17–168)	64 (26–184)	0.76
Plasma creatinine (µmol/L)	73 (57–104)	73 (61–104)	0.86
Troponin (ng/L)	21 (16–46)	17 (16–35)	0.16
N-terminal pro B-type natriuretic peptide (pg/mL)	116 (52–214)	206 (69–893)	0.04
Management during ICU stay			
High-flow nasal oxygen therapy, *n* (%)	29 (64)	32 (44)	0.03
Non invasive ventilation, *n* (%)	1 (2)	3 (4)	0.58
Intubation, *n* (%)	27 (60)	58 (80)	0.02
Corticosteroids, *n* (%)	36 (80)	59 (81)	0.91
Antiviral drugs, *n* (%)	6 (13)	9 (12)	0.87
Tocilizumab, *n* (%)	19 (42)	18 (25)	0.05
Hydroxychloroquine, *n* (%)	3 (7)	5 (7)	0.97
Low-dose thrombophylaxis, *n* (%)	2 (4)	8 (11)	0.22
Enhanced intermediate-dose thrombophylaxis, *n* (%)	36 (80)	52 (71)	0.29
Curative anticoagulation, *n* (%)	7 (16)	18 (25)	0.24
Norepinephrine, *n* (%)	18 (40)	50 (69)	< 0.01
Dobutamine, *n* (%)	0 (0)	1 (1)	0.43
Neuromuscular blocker agents, *n* (%)	21 (47)	51 (70)	0.01
Prone positioning, *n* (%)	30 (67)	57 (78)	0.17
Nitric oxide, *n* (%)	26 (58)	27 (37)	0.03
Venovenous ECMO, *n* (%)	1 (2)	3 (4)	0.58
Renal replacement therapy, *n* (%)	8 (18)	16 (22)	0.59

*n* = 118 patients. Variables are expressed as median (interquartile) or numbers (percentages)

ECMO: extracorporeal membrane oxygenation; FiO_2_: inspired fraction of oxygen; ICU: intensive care unit; PaO_2_: partial arterial pressure of oxygen; PaCO_2_: partial arterial pressure of carbon dioxide; RV: right ventricular; SAPS: simplified acute physiology score; SOFA: sepsis-related organ failure assessment; TTE: transthoracic echocardiography

### RV injury during ICU stay

**Fig. 2:**
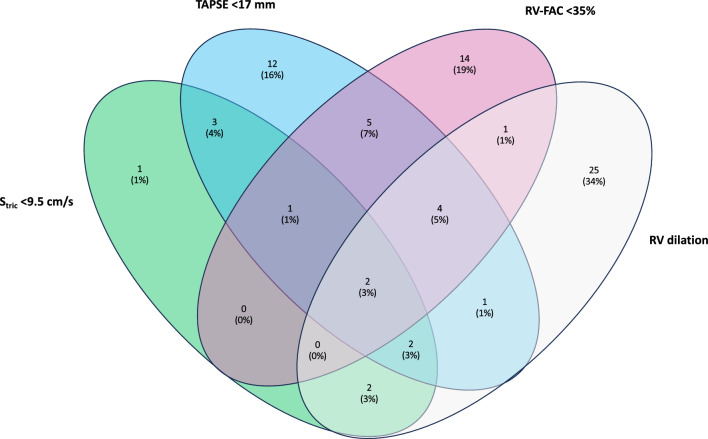
Venn diagram illustrating the different parameters used to diagnose right ventricular (RV) dysfunction in patients with RV injury during intensive care unit stay. TAPSE: tricuspid annular plane systolic excursion; RV-FAC: right ventricular fractional area change; *S*_tric_: systolic tricuspid annular velocity

Overall, 73(62%) patients had at least one RV injury pattern, 12(10%) had LV systolic dysfunction and 23(19%) had LV diastolic dysfunction during one or several TTE examinations during ICU stay. Interobserver variability was 0.98, 0.97, 0.90, 0.93, 0.90, 0.79 and 0.88 for LV ejection fraction, LVEDA, RVEDA, TAPSE, systolic tricuspid annular velocity, RV fractional area change and RVEDA/LVEDA ratio respectively. RV injury was isolated in 82% of cases and combined with left ventricular systolic dysfunction in 18% of cases. The diagnosis of RV dysfunction was based on the RV fractional area change impairment only in 14(19%) patients, on TAPSE impairment only in 12(16%) patients and on systolic tricuspid annular velocity impairment only in 1(1%) patient (Fig. 2). Patients with RV injury were more likely to have cardiovascular risk factors, were more likely to be intubated (80% vs. 60%, *p* = 0.02), were more likely to receive norepinephrine (69% vs. 40%, *p* < 0.01) (Table 1) and had a longer ICU length of stay than those without (Additional file 1: Table S1).

During the first TTE examination on ICU admission, 65(55%) patients had no RV injury, 18(15%) had isolated RV dilation, 28(24%) had RV dysfunction without RV dilation, 5(4%) had RV dysfunction with RV dilation and 2(2%) had ACP (Table 2). When only TAPSE measurement was considered, 75(63%) patients had no RV injury, 20(17%) had isolated RV dilation, 18(15%) had RV dysfunction without RV dilation, 3(3%) had RV dysfunction with RV dilation and 2(2%) had ACP. Patient characteristics, management and outcomes as well as echocardiographic variables according to the RV injury pattern on ICU admission are summarized in Table 2 and Additional file 1: Table S2.

**Table 2:** Echocardiographic variables according to the RV injury pattern on ICU admission

	No RV injury (*n* = 65)	Isolated RV dilatation (*n* = 18)	RV dysfunction without RV dilatation (*n* = 28)	RV dysfunction with RV dilatation (*n* = 5)	Acute cor pulmonale (*n* = 2)	*p*-value
Ventilatory settings at the time of TTE examination						
Standard oxygenation, *n* (%)	0 (0)	1 (6)	0 (0)	0 (0)	0 (0)	< 0.01
High-flow nasal oxygen therapy, *n* (%)	31 (48)	8 (44)	14 (50)	0 (0)	0 (0)	0.18
Non invasive ventilation, *n* (%)	0 (0)	1 (6)	1 (4)	0 (0)	0 (0)	0.49
Intubation, *n* (%)	34 (52)	8 (44)	13 (46)	5 (100)	2 (100)	0.13
Positive end-expiratory pressure (cmH20)	12 (11–15)	11 (7–12)	11 (10–12)	10 (10–12)	16 (12–20)	0.11
Driving pressure (cmH_2_O)	13 (11–14)	13 (11–18)	12 (11–12)	12 (12–13)	13 (12–14)	0.20
Compliance of the respiratory system (mL/cmH2O)	31 (25–38)	27 (20–29)	35 (30–41)	33 (26–33)	37 (32–41)	0.15
Oxygenation variables at the time of TTE examination						
pH	7.45 (7.39–7.50)	7.40 (7.36–7.49)	7.46 (7.39–7.49)	7.33 (7.30–7.37)	7.30 (7.19–7.34)	0.06
FiO_2_ (%)	0.7 (0.5–0.9)	0.6 (0.6–0.8)	0.7 (0.6–0.8)	0.4 (0.3–0.5)	0.8 (0.6–1.0)	0.05
PaO_2_/FiO_2_	116 (80–190)	102 (79–135)	110 (84–188)	247 (159–296)	84 (54–115)	0.04
PaCO_2_ (mmHg)	36 (32–41)	43 (38–47)	36 (32–40)	37 (35–40)	45 (42–48)	0.01
Blood lactate level (mmol/L)	1.1 (0.9–1.3)	1.3 (0.9–1.5)	1.2 (1.0–1.3)	1.1 (0.9–1.8)	0.7 (0.7–0.8)	0.07
Hemodynamic variables at the time of TTE examination						
Heart rate (bpm)	74 (67–83)	73 (69–94)	80 (70–90)	65 (62–97)	130 (103–157)	0.12
Systolic arterial pressure (mmHg)	122 (114–135)	118 (107–127)	124 (113–134)	106 (104–122)	100 (100–101)	0.19
Diastolic arterial pressure (mmHg)	62 (57–70)	57 (53–63)	65 (55–73)	60 (52–72)	57 (50–65)	0.40
Mean arterial pressure (mmHg)	81 (77–93)	77 (72–85)	81 (78–90)	77 (74–93)	57 (48–66)	0.09
Norepinephrine, *n* (%)	24 (37)	5 (28)	12 (43)	3 (60)	2 (100)	0.26
Norepinephrine dosage (µg/kg/min)	0.11 (0.03–0.20)	0.15 (0.07–0.33)	0.17 (0.06–0.24)	0.31 (0.28–0.42)	0.18 (0.05–0.30)	0.26
Echocardiographic variables						
LV ejection fraction (%)	63 (55–68)	68 (58–77)	56 (50–66)	60 (57–62)	70 (58–82)	0.06
Velocity–time integral of the LV outflow tract (cm)	22 (19–24)	21 (18–24)	22 (18–27)	16 (15–18)	24 (18–30)	0.09
E/A ratio	0.90 (0.81–1.14)	1.01 (0.74–1.33)	0.99 (0.81–1.25)	1.10 (1.01–1.14)	0.94 (0.69–1.19)	0.78
e’_septal_ (cm/s)	8.9 (6.8–9.4)	7.5 (6.2–8.6)	7.0 (5.4–8.7)	7.4 (7.1–8.2)	13.3 (11.6–15.0)	0.01
e’_lateral_ (cm/s)	9.8 (8.0–12.7)	9.6 (6.4–10.9)	9.5 (7.0–11.1)	8.4 (7.8–9.1)	13.0 (10.9–15.0)	0.40
E/e’_averaged_	8.00 (6.71–8.91)	8.12 (6.15–8.94)	9.30 (7.59–11.03)	7.46 (5.73–8.14)	4.91 (4.43–5.40)	0.03
TAPSE (mm)	22 (20–26)	23 (20–26)	16 (15–20)	15 (14–18)	19 (18–20)	< 0.01
Systolic tricuspid annular velocity (cm/s)	15 (13–18)	14 (12–16)	13 (10–16)	11 (9–12)	13 (11–14)	0.01
RV FAC (%)	47 (43–57)	49 (44–51)	38 (31–48)	33 (28–33)	33 (31–34)	< 0.01
RV/LV end-diastolic areas ratio	0.24 (0.16–0.47)	0.72 (0.67–0.80)	0.19 (0.15–0.48)	0.83 (0.74–0.85)	0.75 (0.66–0.85)	< 0.01
SPAP (mmHg)	26 (21–40)	31 (30–32)	25 (20–32)	31 (20–39)	N/A	0.47
Paradoxical septal motion, *n* (%)	0 (0)	0 (0)	0 (0)	0 (0)	2 (100)	< 0.01
TAPSE/SPAP (mm/mmHg)*	0.84 (0.57–1.02)	0.74 (0.68–0.85)	0.60 (0.46–0.72)	0.64 (0.46–0.78)	–	0.30
RV FAC/SPAP (%/mmHg)*	1.92 (1.39–2.20)	1.59 (1.25–1.72)	1.70 (0.96–2.45)	1.13 (0.84–1.62)	–	0.05

*n* = 118 patients. Variables are summarized as median (interquartile range) and numbers (percentages)

^*^ SPAP measurement available in 62 patients

ICU: intensive care unit; TTE: transthoracic echocardiography; LV: left ventricular; E: early peak velocity of transmitral flow with pulsed Doppler; A: atrial peak velocity of transmitral flow with pulsed Doppler; e’: early diastolic peak velocity of the mitral annulus with tissue Doppler imaging; TAPSE: tricuspid annular plane systolic excursion; RV FAC: right ventricular fractional area change; SPAP: systolic pulmonary artery pressure

**Fig. 3:**
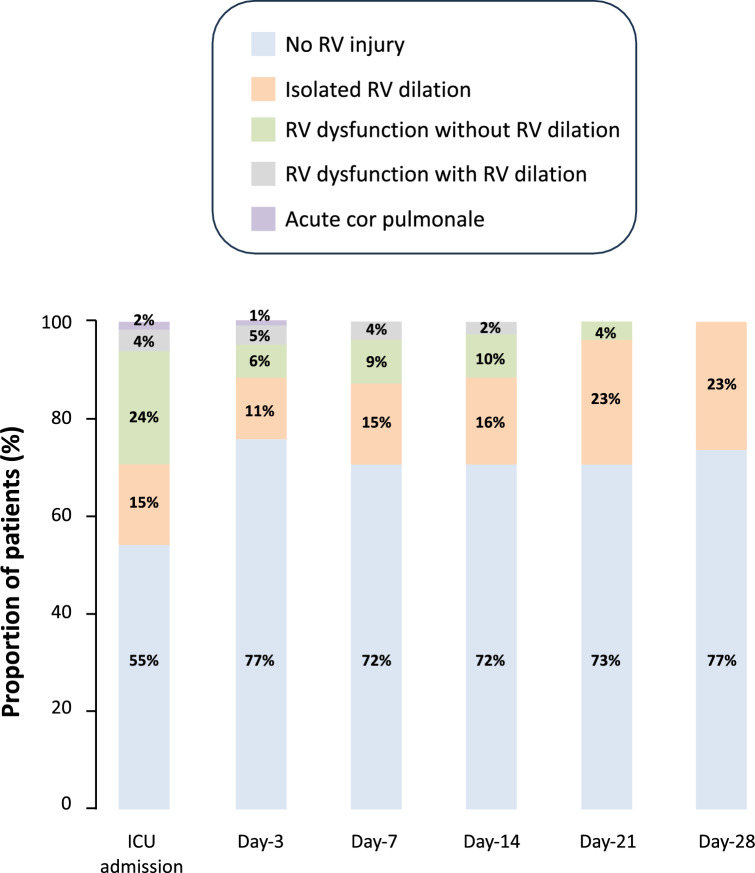
Distribution of the different right ventricular (RV) injury patterns during intensive care unit (ICU) stay

Among the 73 patients with at least one RV injury pattern during one or several TTE examinations during ICU stay, 66(90%) patients experienced a single RV injury pattern and 7(10%) patients experienced several patterns of RV injury: 29(40%) had isolated RV dilation, 39(53%) had RV dysfunction without RV dilation, 10(14%) had RV dysfunction with RV dilation and 2(3%) had ACP (Table 3 and Additional file 1: Tables S3, Fig. 3 and Additional file 1: Fig. S2). When only TAPSE measurement was considered, 58(49%) patients had at least one RV injury pattern during one or several TTE examinations during ICU stay. Among them, 52(90%) experienced a single RV injury pattern and 6(10%) experienced several patterns of RV injury: 30(52%) had isolated RV dilation, 25(43%) had RV dysfunction without RV dilation, 7(12%) had RV dysfunction with RV dilation and 2(3%) had ACP. The median delay of occurrence during ICU stay was 1(1–3) day for RV isolated dilation, 1(1–3) day for RV dysfunction without RV dilation, 2(1–3) days for RV dysfunction with RV dilation and 1(1–1) day for ACP. When pooling all TTE examinations during ICU stay according to the RV injury pattern, patients with the most severe RV injury patterns were all intubated at the time of TTE examination and tended to have higher driving pressure (Additional file 1: Table S4).

**Table 3:** Distribution of the different RV injury patterns during ICU stay

Number of patients	ICU admission	Day-3	Day-7	Day-14	Day-21	Day-28	*p*-value
	118	117	80	43	22	13	–
No RV injury, *n* (%)	65 (55)	90 (77)	58 (72)	31 (72)	16 (73)	10 (77)	< 0.01
Isolated RV dilatation, *n* (%)	18 (15)	13 (11)	12 (15)	7 (16)	5 (23)	3 (23)	0.66
RV dysfunction without RV dilatation, *n* (%)	28 (24)	7 (6)	7 (9)	4 (10)	1 (4)	0 (0)	< 0.01
RV dysfunction with RV dilatation, *n* (%)	5 (4)	6 (5)	3 (4)	1 (2)	0 (0)	0 (0)	0.75
Acute cor pulmonale, *n* (%)	2 (2)	1 (1)	0 (0)	0 (0)	0 (0)	0 (0)	0.77

Variables are expressed as numbers (percentages)

ICU: intensive care unit; RV: right ventricular

**Fig. 4:**
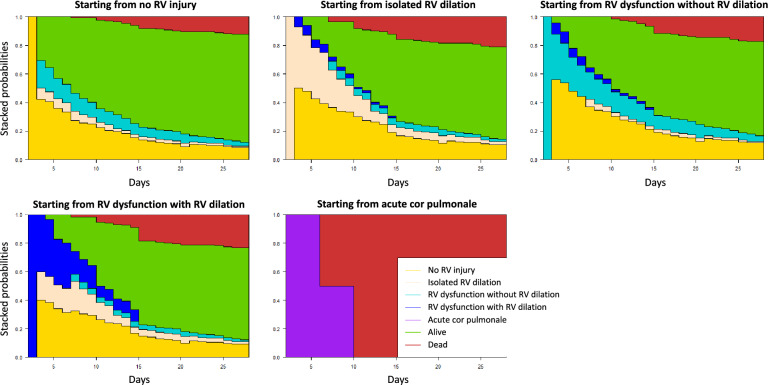
Cumulative probability of being at a state at a given time during intensive care unit (ICU) stay according to the right ventricular (RV) injury pattern on ICU admission

Regardless of the RV injury pattern, the number of patients with de novo RV injury decreased over time, no patient developed de novo RV injury after Day-14 and 20(31%) patients without RV injury on ICU admission developed RV injury during ICU stay (Additional file 1: Table S5, Fig. S3, Fig. 4). On ICU admission, these patients were more frequently intubated (75 vs. 42%, *p* = 0.03) and were more frequently ventilated with a PEEP level > 12 cmH2O (71 vs. 30%, *p* = 0.03) than those who did not developed RV injury during ICU stay. There was no difference in ventilatory settings, respiratory mechanics, oxygenation and hemodynamic variables at the time of TTE examination between RV injury diagnosis and the previous TTE examination in patients with no RV injury on ICU admission who developed RV injury during ICU stay (Additional file 1: Table S6).

### RV injury and mortality rate

The Day-28 mortality rate was higher in patients with RV injury than in those without (27 vs. 7%, *p* < 0.01) (Additional file 1: Tables S1, 2, Figure S4A). At multivariate analysis, the combination of RV dysfunction with RV dilation or ACP (aHR = 3.18 95% CI(1.16–8.74), *p* = 0.03), age > 65 years, cardiovascular chronic disease, immunosuppression and PaCO_2_ > 45 mmHg at the time of TTE examination were associated with Day-28 mortality (Additional file 1: Table S7, Fig. S4B). In mixed effect logistic regression model, RV injury was no longer associated with mortality (Additional file 1: Table S8).

## Discussion

In this prospective cohort of 118 critically ill patients with COVID-19 and systematic and content-standardized echocardiographic follow-up during ICU stay at predetermined times, 62% of patients had RV injury during ICU stay, no patient developed de novo RV injury after Day-14 regardless of the RV injury pattern and 10% of patients with RV injury experienced several patterns of RV injury during ICU stay. Only the combination of RV dysfunction with RV dilation or ACP was associated with Day-28 mortality.

The majority of patients experienced RV injury during ICU stay and the most frequent RV injury pattern both on ICU admission and during ICU stay was RV dysfunction without RV dilation. Other studies found that isolated RV dilation was the most frequent RV injury pattern on ICU admission [8, 9]. This may be explained by the different definitions of RV injury used and the different echocardiographic parameters used to assess RV dysfunction. It has been shown in COVID-19 patients with ARDS that the proportion of patients considered with RV injury varied according to the echocardiographic parameter of RV systolic function that was used, with a higher proportion of patients with RV injury identified when using the RV fractional area change than when using TAPSE or the systolic tricuspid annular velocity, suggesting that RV injury in COVID-19 patients is related to a RV radial impairment with sparing of longitudinal RV function [3]. We chose to combine the three echocardiographic parameters of RV systolic function for the diagnosis of RV dysfunction, as it has been shown that TAPSE could be not sensitive enough to detect RV involvement [3], especially in patients mechanically ventilated [23]. When only TAPSE measurement was considered, we confirmed that isolated RV dilation was the most frequent RV injury pattern both on admission and during ICU stay. Interestingly, RV injury was associated with LV dysfunction in 18% of cases, confirming the previous findings, which showed a potential impact of LV systolic function on TAPSE, as illustrated by an increase in the proportion of patients with RV systolic dysfunction with the severity of LV dysfunction and by a correlation between TAPSE and LV ejection fraction measurements [6]. Finally, LV filling pressure was not elevated overall, which is expected in these patients with ARDS, although approximately 50% of them had a history of arterial hypertension and 10% of them a history of coronary artery disease.

To our knowledge, this is the first study assessing RV injury with systematic and content-standardized echocardiographic follow-up during ICU stay at predetermined times not clinically driven, allowing us to analyze the patient trajectories in terms of RV injury during ICU stay. In agreement with previous studies [5, 6, 8, 9], most patients had no RV injury on ICU admission. Patients without RV injury on ICU admission who subsequently developed RV injury were more frequently intubated and ventilated with high PEEP level on ICU admission. Therefore, RV injury occurrence during ICU stay may be related to the severity of respiratory mechanics and oxygenation impairment. Nevertheless, we found no worsening of respiratory mechanics and oxygenation between RV injury diagnosis and preceding echocardiography examination in patients without RV injury on ICU admission who subsequently developed RV injury, conversely to Evrard and colleagues [8]. Such discrepancy may be explained by the fact that in our study RV injury was diagnosed during TTE examinations performed at predefined times according to standardized echocardiographic follow-up and not necessarily during TTE examinations at the time of patients' clinical deterioration. Moreover, RV injury occurred early in the ICU stay and the number of patients with de novo RV injury decreased over time with no patient developing de novo RV injury after Day-14, regardless of the RV injury pattern. Others found that patients could develop RV injury during the first three weeks after ICU admission [8]. Nevertheless, only weekly echocardiographic examinations were performed after the initial examination, which may overestimate the delay in RV injury development. Finally, 10% of patients with RV injury experienced several patterns of RV injury during ICU stay, confirming that patients can move from one RV injury pattern to another with some patients experiencing all RV injury patterns during ICU stay [11]. All these results highlight the importance of echocardiographic follow-up to assess RV injury during ICU stay in patients with ARDS, regardless of whether there has been any appreciable clinical change and despite normal RV function on ICU admission.

The previous studies assessed the prognostic value of RV injury in non-critically ill [24–26] and in critically ill [4–6, 8–12] COVID-19 patients with contrasting findings. We hypothesized that RV injury was associated with patient mortality, irrespective of the timing of its development and we found that only the combination of RV dysfunction with RV dilation or ACP was associated with Day-28 mortality. Owing to a lack of power and the fact that such an analysis does not take into account at which time echocardiography was performed, RV injury was no longer associated with mortality in mixed effect logistic regression model. However, our results combined with those of previous studies suggest that the prognostic value of RV injury in COVID-19 patients may depend on the severity of RV injury, as RV dilation with systolic impairment [4] or ACP [6, 8, 9, 11], but not isolated RV dilation or RV dysfunction without RV dilation [4–6, 9] were found to be independently associated with mortality. Interestingly, the association between the most severe pattern of RV injury and patient mortality was found regardless of the RV injury definition, including visual assessment of RV function [6], echocardiographic assessment with or without RV strain measurements [4, 5, 9] or using a definition combining RV dilation and systemic venous congestion [8, 11]. All these results may suggest that RV systolic dysfunction is probably not per se associated with patient mortality and the hypothesis of a gradient of severity from isolated RV dilation to ACP. Physiologically, RV dilatation may be initially considered as a functional adaptative mechanism to maintain cardiac output despite an increase in RV afterload, according to Frank-Starling’s law. If decompensatory factors persist, a more marked RV dilation with RV systolic dysfunction may be observed and may lead to a decrease of left ventricular distensibility and filling due to the phenomenon of ventricular interdependence [27–29] and in most severe patients, to ACP reflecting marked uncoupling between the RV and pulmonary artery [30], both participating to hemodynamic failure and poor patient prognosis [31]. Nevertheless, the combination of RV dysfunction with RV dilation remains seldom as it reflects the worst uncoupling between RV and pulmonary artery, as illustrated in our cohort by a decrease in RV coupling metrics in these patients [19–21].

Conversely to previous studies which found a prevalence of ACP from 20% [1, 4–6, 9] to 37% [11], we unexpectedly found in our cohort a very low prevalence of ACP, while patients with ACP were more severe and all were intubated and ventilated with high PEEP level at the time of ACP diagnosis. Moreover, ACP was always diagnosed during the first TTE examination on ICU admission and none of the patients developed ACP during ICU stay. These unexpected findings can be explained as follows. First, an enhanced intermediate-dose thrombophylaxis was administered in 80% of patients, which may have reduced the incidence of thrombo-embolic events such as pulmonary embolism during ICU stay. Second, the decrease in invasive mechanical ventilation over the different pandemic waves [32], which has been shown to be a risk factor of cardiac injury in COVID-19 patients [13]. Although the proportion of patients who were mechanically ventilated during ICU stay was similar in our cohort and in previous studies, only 60% of patients were intubated at the time of TTE examination in the first two weeks after ICU admission, when patients had de novo RV injury, which was lower than in previous studies [1, 4, 8, 9], therefore decreasing the potential impact of mechanical ventilation on RV afterload. Third, we performed only TTE examinations in our study when other studies combined TTE and transesophageal echocardiography [6, 8, 11] and it cannot be excluded that the use of TTE echocardiography only during ICU stay may have contributed to underestimating the prevalence of ACP.

We acknowledge some limitations to our study. First, we did not evaluate RV function by using strain measurements because of the difficulty to obtain reliable measurements with TTE in ICU patients, while it has been demonstrated that strain rather than conventional parameters could be associated with mortality in patients with COVID-19 [24, 25, 33, 34]. Second, 21% of patients were excluded because of incomplete echocardiographic follow-up due to failure to respect the timing of TTE examinations and/or the unavailability of experienced operators. Nevertheless, this made it possible to obtain the most reliable echocardiographic follow-up possible. Third, we could not use a definition of RV injury including elevated central venous pressure reflecting systemic venous congestion [23], as its measurement was not part of standard care in the participated ICUs. Fourth, tricuspid regurgitation was present in 60% of TTE examinations, but its severity was not specifically assessed, preventing evaluation of its impact on functional RV systolic parameters. Fifth, given the very low proportion of patients with ACP, it was not possible to analyze the prognostic value of this RV injury pattern in isolation at multivariate analysis. Sixth, while RV injury is likely to be related to increased RV afterload in patients with COVID-19 [8, 13], there was no difference in the respiratory characteristics of patients on ICU admission, regardless of the different RV injury patterns. This lack of difference may be mainly due to a lack of power given the relatively small number of patients for each RV injury patterns and it was therefore not possible to further interpret this finding, as with other physiological findings on ICU admission such as oxygenation parameters, to avoid overinterpretation. Nevertheless, when pooling all TTE examinations during ICU stay according to the RV injury pattern, patients with the most severe RV injury patterns were all intubated at the time of TTE examination and tended to have poorer respiratory mechanics. Seventh, the potential chronicity of some echocardiographic abnormalities could not be excluded with certainty as some patients had no or no available results of a previous cardiac evaluation.

## Conclusion

In critically ill patients with COVID-19, while most did not have RV injury on ICU admission, RV injury was frequent during ICU stay and occurred within the first two weeks after ICU admission, highlighting the importance of echocardiographic follow-up to assess RV injury during ICU stay in patients with ARDS, regardless of whether there has been any appreciable clinical change and despite normal RV function on ICU admission. RV injury was most often isolated and the most frequent RV injury pattern both on ICU admission and during ICU stay was RV dysfunction without RV dilation. Only the combination of RV dysfunction with RV dilation or ACP could potentially be associated with Day-28 mortality.

### Supplementary Information

**Additional file 1: Figure S2:** Alluvial plot with distribution of patients depending on their right ventricular (RV) injury pattern and status (alive or dead) from intensive care unit (ICU) admission to Day-28. 1. No RV injury (blue lines). 2. Isolated RV dilatation (green lines). 3. RV dysfunction without RV dilatation (yellow lines). 4. RV dysfunction with RV dilatation (red lines). 5. Acute cor pulmonale (black lines). 6. Alive. 7. Dead. Among the 65 patients without RV injury on ICU admission, 7(11%) experienced isolated RV dilation, 8(12%) experienced RV dysfunction without dilation, 5(8%) experienced RV dysfunction with RV dilation and no patient experienced acute cor pulmonale during ICU stay. Among the 18 patients with isolated RV dilation on ICU admission, 1(6%) experienced RV dysfunction with RV dilation and no patient experienced RV dysfunction without dilation or acute cor pulmonale during ICU stay. Among the 28 patients with RV dysfunction without dilation on ICU admission, 1(4%) experienced isolated RV dilation, 2(7%) experienced RV dysfunction with RV dilation and no patient experienced acute cor pulmonale during ICU stay. Among the 5 patients with RV dysfunction with RV dilation on ICU admission, 2(40%) experienced isolated RV dilation, 1(20%) experienced RV dysfunction without RV dilation and no patient experienced acute cor pulmonale during ICU stay. Among the 2 patients with acute cor pulmonale on ICU admission, 1(50%) experienced isolated RV dilation, 2(100%) experienced RV dysfunction with RV dilation and no patient experienced RV dysfunction without RV dilation during ICU stay. **Figure S3:** Cumulative incidence of the different right ventricular (RV) injury patterns during intensive care unit stay. **Figure S4:** *Panel A*: Cumulative incidence of Day-28 mortality according to the most severe right ventricular (RV) injury pattern during intensive care unit (ICU) stay (*p*-value according to log rank test). *Panel B*: Risk factors for Day-28 mortality. aHR: adjusted hazard ratio, CI: confidence interval, PaCO_2_: partial arterial pressure of carbon dioxide, RV: right ventricular. *Cardiovascular chronic disease = coronary artery disease + stroke + chronic heart failure. **Table S1.** Patient outcomes according to the presence of RV injury during ICU stay. **Table S2.** Patient characteristics, management and outcomes according to the RV injury pattern on ICU admission. **Table S3.** Echocardiographic variables during ICU stay in the whole population. **Table S4.** Ventilatory settings, oxygenation and hemodynamic variables when pooling all TTE examinations during ICU stay according to the RV injury pattern. **Table S5.** Number of patients with de novo RV injury during ICU stay. **Table S6.** Ventilatory settings, oxygenation and hemodynamic variables at TTE examination before and at the time of RV injury diagnosis in patients without RV injury on ICU admission. **Table S7.** Risk factors for Day-28 mortality. Table S8. Mixed effect logistic regression to assess the impact of RV injury on the Day-28 mortality rate.

**Additional file 2: Figure S1.** Echocardiographic loops illustrating the four different right ventricular (RV) injury patterns. *Panel A*: isolated RV dilation (apical 4-chamber view). *Panel B:* RV dysfunction without RV dilation (apical 4-chamber view). *Panel C*: RV dysfunction with RV dilation (apical 4-chamber view). Panel D: acute cor pulmonale with paradoxical septal motion (apical 4-chamber view).

## Data Availability

The datasets used and/or analyzed during the current study are available from the corresponding author on reasonable request.

## Abbreviations

ACP: Acute cor pulmonale
ARDS: Acute respiratory distress syndrome
COVID-19: Coronavirus disease 19
ICU: Intensive care unit
LVEDA: Left ventricular end-diastolic area
PaCO_2_: Partial arterial pressure of carbon dioxide
PEEP: Positive end-expiratory pressure
RV: Right ventricular
RVEDA: Right ventricular end-diastolic area
TAPSE: Tricuspid annular plane systolic excursion
TTE: Transthoracic echocardiography
