# Supplementary material for: Right ventricular injury in critically ill patients with COVID-19: a descriptive study with standardized echocardiographic follow-up
Source: Ann Intensive Care. 2024 Jan 23;14:14. doi: 10.1186/s13613-024-01248-8 (PMC10805901; doi:10.1186/s13613-024-01248-8)
Supplement: Supplementary file 1 — Additional file 1: Figure S2: Alluvial plot with distribution of patients depending on their right ventricular (RV) injury pattern and status (alive or dead) from intensive care unit (ICU) admission to Day-28. 1. No RV injury (blue lines). 2. Isolated RV dilatation (green lines). 3. RV dysfunction without RV dilatation (yellow lines). 4. RV dysfunction with RV dilatation (red lines). 5. Acute cor pulmonale (black lines). 6. Alive. 7. Dead. Among the 65 patients without RV injury on ICU admission, 7(11%) experienced isolated RV dilation, 8(12%) experienced RV dysfunction without dilation, 5(8%) experienced RV dysfunction with RV dilation and no patient experienced acute cor pulmonale during ICU stay. Among the 18 patients with isolated RV dilation on ICU admission, 1(6%) experienced RV dysfunction with RV dilation and no patient experienced RV dysfunction without dilation or acute cor pulmonale during ICU stay. Among the 28 patients with RV dysfunction without dilation on ICU admission, 1(4%) experienced isolated RV dilation, 2(7%) experienced RV dysfunction with RV dilation and no patient experienced acute cor pulmonale during ICU stay. Among the 5 patients with RV dysfunction with RV dilation on ICU admission, 2(40%) experienced isolated RV dilation, 1(20%) experienced RV dysfunction without RV dilation and no patient experienced acute cor pulmonale during ICU stay. Among the 2 patients with acute cor pulmonale on ICU admission, 1(50%) experienced isolated RV dilation, 2(100%) experienced RV dysfunction with RV dilation and no patient experienced RV dysfunction without RV dilation during ICU stay. Figure S3: Cumulative incidence of the different right ventricular (RV) injury patterns during intensive care unit stay. Figure S4: Panel A: Cumulative incidence of Day-28 mortality according to the most severe right ventricular (RV) injury pattern during intensive care unit (ICU) stay (p-value according to log rank test). Panel B: Risk factors for Day-28 mortality. aHR: [file 13613_2024_1248_MOESM1_ESM.docx]

**Right ventricular injury in critically ill patients with COVID-19 : a descriptive study with standardized echocardiographic follow-up**

Mathieu JOZWIAK, MD PhD^1,2,3^ ; Claire DUPUIS, MD PhD^4,5^ ; Pierre DENORMANDIE, MD^1^ ; Didac AURENCHE MATEU, MD^6^ ; Jean LOUCHET, MD^6^ ; Nathan HEME, MD^6^ ; Jean-Paul MIRA, MD PhD^1,2^ ; Denis DOYEN, MD PhD^3,6^ ; Jean DELLAMONICA, MD PhD^3,6^

1 : Service de Médecine Intensive Réanimation, Hôpitaux Universitaires Paris Centre, Hôpital Cochin, Assistance Publique – Hôpitaux de Paris, 27 Rue du Faubourg Saint Jacques, 75014 Paris, France

2 : Université Paris Cité, France

3 : UR2CA - Unité de Recherche Clinique Côte d'Azur, Université Côte d’Azur, Nice, France

4 : Service de Médecine Intensive Réanimation, Centre Hospitalier Universitaire de Clermont-Ferrand, Hôpital Gabriel Montpied, 58 rue Montalembert, 63000 Clermont-Ferrand, France

5 : IAME Université Paris Cité, U 1137, 75018, Paris France

6 : Service de Médecine Intensive Réanimation, Centre Hospitalier Universitaire de Nice, Hôpital l’Archet 1, 151 rue saint Antoine de Ginestière, 06200 Nice, France

***Corresponding author***

Mathieu JOZWIAK, MD, PhD

Hôpital Cochin, APHP

Service de Médecine Intensive Réanimation

27 Rue du Faubourg Saint Jacques, 75014 Paris, France

[jozwiak.m@chu-nice.fr](mailto:jozwiak.m@chu-nice.fr)

**Figures legends**

**Figure S1**

Echocardiographic loops illustrating the four different right ventricular (RV) injury patterns. *Panel A*: isolated RV dilation (apical 4-chamber view). *Panel B:* RV dysfunction without RV dilation (apical 4-chamber view). *Panel C*: RV dysfunction with RV dilation (apical 4-chamber view). Panel D: acute cor pulmonale with paradoxical septal motion (apical 4-chamber view).

**Figure S2**

Alluvial plot with distribution of patients depending on their right ventricular (RV) injury pattern and status (alive or dead) from intensive care unit (ICU) admission to Day-28. 1. No RV injury (blue lines). 2. Isolated RV dilatation (green lines). 3. RV dysfunction without RV dilatation (yellow lines). 4. RV dysfunction with RV dilatation (red lines). 5. Acute cor pulmonale (black lines). 6. Alive. 7. Dead.

Among the 65 patients without RV injury on ICU admission, 7(11%) experienced isolated RV dilation, 8(12%) experienced RV dysfunction without dilation, 5(8%) experienced RV dysfunction with RV dilation and no patient experienced acute cor pulmonale during ICU stay. Among the 18 patients with isolated RV dilation on ICU admission, 1(6%) experienced RV dysfunction with RV dilation and no patient experienced RV dysfunction without dilation or acute cor pulmonale during ICU stay. Among the 28 patients with RV dysfunction without dilation on ICU admission, 1(4%) experienced isolated RV dilation, 2(7%) experienced RV dysfunction with RV dilation and no patient experienced acute cor pulmonale during ICU stay. Among the 5 patients with RV dysfunction with RV dilation on ICU admission, 2(40%) experienced isolated RV dilation, 1(20%) experienced RV dysfunction without RV dilation and no patient experienced acute cor pulmonale during ICU stay. Among the 2 patients with acute cor pulmonale on ICU admission, 1(50%) experienced isolated RV dilation, 2(100%) experienced RV dysfunction with RV dilation and no patient experienced RV dysfunction without RV dilation during ICU stay.

**Figure S3**

Cumulative incidence of the different right ventricular (RV) injury patterns during intensive care unit stay.

**Figure S4**

*Panel A*: Cumulative incidence of Day-28 mortality according to the most severe right ventricular (RV) injury pattern during intensive care unit (ICU) stay (p-value according to log rank test).

*Panel B*: Risk factors for Day-28 mortality. aHR: adjusted hazard ratio, CI: confidence interval, PaCO_2_: partial arterial pressure of carbon dioxide, RV: right ventricular. *Cardiovascular chronic disease = coronary artery disease + stroke + chronic heart failure.

| **Table S1. Patient outcomes according to the presence of RV injury during ICU stay.** | | | |
| --- | --- | --- | --- |
|  | **No RV injury**  **(n=45)** | **RV injury**  **(n=73)** | **p-value** |
| **Outcomes** |  |  |  |
|  |  |  |  |
| Pulmonary embolism, n (%) | 4 (9) | 5 (7) | 0.69 |
| Deep venous thrombosis, n (%) | 6 (13) | 8 (11) | 0.70 |
| Acute coronary syndrome, n (%) | 2 (4) | 3 (4) | 0.93 |
| Duration of invasive mechanical ventilation (days) | 15 (4-36) | 14 (8-30) | 0.77 |
| ICU length of stay (days) | 8 (5-25) | 14 (8-25) | 0.03 |
| ICU mortality rate, n (%) | 5 (11) | 18 (25) | 0.07 |
| Day-28 mortality rate, n (%) | 3 (7) | 20 (27) | <0.01 |
| Day-90 mortality rate, n (%) | 5 (11) | 22 (30) | 0.02 |
| Overall mortality rate, n (%) | 6 (13) | 24 (33) | 0.02 |
| *n=118 patients. Variables are expressed as median (interquartile) or numbers (percentages).*  *ICU: intensive care unit; RV: right ventricular.* | | | |

| **Table S2. Patient characteristics, management and outcomes according to the RV injury pattern on ICU admission.** | | | | | | |
| --- | --- | --- | --- | --- | --- | --- |
|  | **No RV injury**  **(n=65)** | **Isolated RV dilatation**  **(n=18)** | **RV dysfunction without RV dilatation**  **(n=28)** | **RV dysfunction with RV dilatation**  **(n=5)** | **Acute cor pulmonale (n=2)** | **p-value** |
| **Clinical characteristics** |  |  |  |  |  |  |
|  |  |  |  |  |  |  |
| Age (years) | 64 (57-71) | 68 (62-73) | 67 (59-74) | 67 (62-73) | 62 (47-78) | 0.40 |
| Gender (male), n (%) | 48 (74) | 10 (56) | 20 (71) | 4 (80) | 2 (100) | 0.50 |
| SAPS-2 score | 39 (31-59) | 51 (36-76) | 41 (32-51) | 72 (63-84) | 63 (44-83) | 0.02 |
| SOFA score on ICU admission | 6 (3-10) | 7 (5-9) | 6 (3-10) | 10 (9-15) | 14 (13-16) | 0.04 |
| Body mass index (kg/m²) | 28 (26-31) | 28 (23-30) | 29 (26-31) | 28 (26-29) | 33 (27-38) | 0.71 |
| Obesity, n (%) | 20 (31) | 6 (33) | 12 (43) | 1 (20) | 1 (50) | 0.75 |
| Arterial hypertension, n (%) | 25 (38) | 14 (78) | 15 (54) | 3 (60) | 1 (50) | 0.05 |
| Diabete mellitus, n (%) | 15 (23) | 5 (28) | 13 (46.43) | 2 (40) | 0 (0) | 0.18 |
| Dyslipidemia, n (%) | 12 (18) | 10 (56) | 7 (25) | 2 (40) | 0 (0) | 0.02 |
| Smokers, n (%) | 10 (15) | 3 (17) | 4 (14.29) | 0 (0) | 0 (0) | 0.85 |
| Coronary artery disease, n (%) | 6 (9) | 2 (11) | 2 (7.14) | 1 (20) | 0 (0) | 0.89 |
| Stroke, n (%) | 2 (3) | 1 (6) | 3 (10.71) | 0 (0) | 0 (0) | 0.60 |
| Chronic heart failure, n (%) | 2 (3) | 1 (6) | 0 (0) | 1 (20) | 0 (0) | 0.24 |
| Chronic respiratory disease, n (%) | 0 (0) | 2 (11) | 2 (7) | 0 (0) | 1 (50) | <0.01 |
| Chronic kidney disease, n (%) | 6 (9.23) | 4 (22) | 9 (32) | 1 (20) | 0 (0) | 0.09 |
| Immunosuppression, n (%) | 8 (12.31) | 6 (33) | 8 (29) | 2 (40) | 0 (0) | 0.12 |
| Renin-Angiotensin System Blockers, n (%) | 22 (33.85) | 11 (61) | 12 (43) | 1 (20) | 0 (0) | 0.16 |
| Time from onset of symptoms to ICU admission (days) | 9 (7-12) | 7 (5-11) | 9 (5-11) | 7 (6-7) | 14 (8-20) | 0.08 |
|  |  |  |  |  |  |  |
| **Biological variables on ICU admission**  **with the first TTE examination on ICU admission** |  |  |  |  |  |  |
|  |  |  |  |  |  |  |
| Leukocytes (G/L) | 8.5 (7.0-11.6) | 7.6 (6.0-10.9) | 8.4 (6.9-10.3) | 10.8 (8.5-12.8) | 18.5 (18.3-18.6) | 0.14 |
| Neutrophils (G/L) | 7.4 (6.1-10.1) | 6.2 (5.0-8.7) | 7.3 (5.7-9.1) | 7.3 (7.2-10.9) | 17.4 (17.1-17.8) | 0.13 |
| Platelet count (G/L) | 238 (186-292) | 215 (149-317) | 222 (174-266) | 306 (225-391) | 212 (154-271) | 0.67 |
| Lymphocytes (G/L) | 0.7 (0.5-0.9) | 0.6 (0.4-0.8) | 0.7 (0.5-0.8) | 1.2 (0.7-1.3) | 0.2 (0.1-0.2) | 0.10 |
| Fibrinogen (g/L) | 6.2 (5.2-7.6) | 7.0 (5.5-7.8) | 5.7 (5.1-7.5) | 6.9 (6.6-7.6) | 4. (2.1-6.1) | 0.39 |
| D-Dimers (µg/L) | 1033 (670-2255) | 1743 (918-2963) | 1187 (652-1519) | 1022 (797-1156) | 12242 (6682-17802) | 0.13 |
| Protein C reactive (mg/L) | 122 (77-246) | 127 (91-232) | 136 (76-208) | 144 (112-180) | 243 (180-307) | 0.79 |
| Procalcitonin (ng/L) | 0.26 (0.13-0.74) | 0.28 (0.14-0.76) | 0.27 (0.08-1.36) | 0.23 (0.20-0.45) | 7.05 (0.26-13.83) | 0.87 |
| Ferritin (ng/mL) | 1075 (591-1649) | 2003 (1005-2940) | 891 (613-3073) | 877 (779-2087) | 808 (808-808) | 0.38 |
| Interleukin-6 (pg/mL) | 68 (17-168) | 49 (15-130) | 79 (40-316) | 36 (24- 99) | 232 (197-268) | 0.15 |
| Plasma creatinine (µmol/L) | 73 (57-104) | 69 (49-104) | 78 (66-115) | 86 (66-88) | 521 (82-960) | 0.57 |
| Troponin (ng/L) | 17 (16-42) | 19 (10-45) | 17 (16-38) | 22 (15-35) | 243 (55-431) | 0.33 |
| N-terminal pro B-type natriuretic peptide (pg/mL) | 107 (52-320) | 863 (276-4270) | 87 (52-237) | 668 (242-2480) | 698 (206-1189) | <0.01 |
|  |  |  |  |  |  |  |
| **Management during ICU stay** |  |  |  |  |  |  |
|  |  |  |  |  |  |  |
| High-flow nasal oxygen therapy, n (%) | 36 (55) | 9 (50) | 15 (54) | 1 (20) | 0 (0) | 0.34 |
| Non invasive ventilation, n (%) | 1 (2) | 2 (11) | 1 (4) | 0 (0) | 0 (0) | 0.38 |
| Intubation, n (%) | 45 (69) | 14 (78) | 19 (68) | 5 (100) | 2 (100) | 0.48 |
| Corticosteroids, n (%) | 52 (80) | 18 (100) | 21 (75) | 3 (60) | 1 (50) | 0.12 |
| Antiviral drugs, n (%) | 10 (15) | 0 (0) | 4 (14) | 0 (0) | 1 (50) | 0.18 |
| Tocilizumab, n (%) | 23 (35) | 0 (0) | 14 (50) | 0 (0) | 0 (0) | <0.01 |
| Hydroxychloroquine, n (%) | 6 (9) | 0 (0) | 2 (7) | 0 (0) | 0 (0) | 0.66 |
| Low-dose thrombophylaxis, n (%) | 7 (11) | 0 (0) | 2 (7) | 1 (20) | 0 (0) | 0.52 |
| Enhanced intermediate-dose thrombophylaxis, n (%) | 52 (80) | 12 (67) | 21 (75) | 3 (60) | 0 (0) | 0.09 |
| Curative anticoagulation, n (%) | 11 (17) | 5 (28) | 6 (21) | 1 (20) | 2 (100) | 0.07 |
| Norepinephrine, n (%) | 35 (54) | 12 (67) | 15 (54) | 4 (80) | 2 (100) | 0.45 |
| Dobutamine, n (%) | 1 (2) | 0 (0) | 0 (0) | 0 (0) | 0 (0) | 0.94 |
| Neuromuscular blocker agents, n (%) | 39 (60) | 12 (67) | 17 (61) | 2 (40) | 2 (100) | 0.65 |
| Prone positioning, n (%) | 47 (72) | 13 (72) | 23 (82) | 2 (40) | 2 (100) | 0.31 |
| Nitric oxide, n (%) | 34 (52) | 1 (6) | 17 (61) | 0 (0) | 1 (50) | <0.01 |
| Venovenous ECMO, n (%) | 2 (3) | 1 (6) | 1 (4) | 0 (0) | 0 (0) | 0.97 |
| Renal replacement therapy, n (%) | 12 (18) | 3 (17) | 8 (29) | 0 (0) | 1 (50) | 0.43 |
|  |  |  |  |  |  |  |
| **Outcomes** |  |  |  |  |  |  |
|  |  |  |  |  |  |  |
| Pulmonary embolism, n (%) | 5 (8) | 2 (11) | 1 (4) | 0 (0) | 1 (50) | 0.17 |
| Deep venous thrombosis, n (%) | 10 (15) | 0 (0) | 2 (7) | 0 (0) | 2 (100) | <0.01 |
| Acute coronary syndrome, n (%) | 4 (6) | 0 (0) | 1 (4) | 0 (0) | 0 (0) | 0.79 |
| Duration of invasive mechanical ventilation (days) | 14 (5-34) | 11 (7-38) | 20 (11-32) | 8 (4-8) | 7 (6-9) | 0.60 |
| ICU length of stay (days) | 11 (6-25) | 12 (10-26) | 15 (6-25) | 12 (10-17) | 8 (6-10) | 0.76 |
| ICU mortality rate, n (%) | 8 (12) | 8 (44) | 5 (18) | 0 (0) | 2 (100) | <0.01 |
| Day-28 mortality rate, n (%) | 6 (9) | 7 (39) | 7 (25) | 1 (20) | 2 (100) | <0.01 |
| Day-90 mortality rate, n (%) | 9 (14) | 8 (44) | 7 (25) | 1 (20) | 2 (100) | 0.01 |
| Overall mortality rate, n (%) | 10 (15) | 10 (56) | 7 (25) | 1 (20) | 2 (100) | <0.01 |
|  |  |  |  |  |  |  |
| *n=118 patients. Variables are expressed as median (interquartile) or numbers (percentages).*  *ECMO: extracorporeal membrane oxygenation; FiO_2_: inspired fraction of oxygen; ICU: intensive care unit; PaO_2_: partial arterial pressure of oxygen; PaCO_2_: partial arterial pressure of carbon dioxide; RV: right ventricular; SAPS: simplified acute physiology score; SOFA: sepsis-related organ failure assessment; TTE: transthoracic echocardiography.* | | | | | | |

| Table S3. Echocardiographic variables during ICU stay in the whole population. | | | | | | | |
| --- | --- | --- | --- | --- | --- | --- | --- |
|  | **ICU admission** | **Day-3** | | **Day-7** | **Day-14** | **Day-21** | **Day-28** |
|  |  | |  |  |  |  |  |
| Number of patients | 118 | | 117 | 80 | 43 | 22 | 13 |
|  |  | |  |  |  |  |  |
| Ventilatory settings at the time of TTE examination |  | |  |  |  |  |  |
|  |  | |  |  |  |  |  |
| Standard oxygenation, n (%) | 1 (1) | | 4 (3) | 1 (1) | 2 (4) | 2 (9) | 1 (8) |
| High-flow nasal oxygen therapy, n (%) | 53 (45) | | 38 (33) | 23 (28) | 2 (4) | 0 (0) | 0 (0) |
| Non invasive ventilation, n (%) | 2 (2) | | 2 (2) | 2 (2) | 0 (0) | 0 (0) | 0 (0) |
| Intubation, n (%) | 62 (52) | | 73 (62) | 54 (67) | 39 (92) | 20 (91) | 12 (92) |
| Positive end-expiratory pressure (cmH20) | 12 (10-14) | | 12 (10-14) | 12 (9-14) | 10 (8-12) | 8 (8-10) | 8 (6-8) |
| Driving pressure (cmH2O) | 13 (11-14) | | 11 (8-15) | 11 (8-14) | 14 (10-16) | 11 (9-18) | 9 (6-14) |
| Compliance of the respiratory system (mL/cmH2O) | 31 (27-38) | | 35 (26-48) | 33 (27-43) | 31 (24-43) | 37 (23-42) | 52 (30-75) |
|  |  | |  |  |  |  |  |
| Hemodynamic variables at the time of TTE examination |  | |  |  |  |  |  |
|  |  | |  |  |  |  |  |
| Heart rate (bpm) | 76 (68-86) | | 78 (61-96) | 85 (66-96) | 87 (76-107) | 92 (78-102) | 101 (97-110) |
| Systolic arterial pressure (mmHg) | 122 (109-135) | | 120 (108-137) | 123 (104-130) | 122 (109-140) | 131 (116-135) | 134 (119-145) |
| Diastolic arterial pressure (mmHg) | 61 (54-70) | | 60 (52-66) | 58 (52-65) | 60 (51-65) | 58 (51-63) | 59 (54-64) |
| Mean arterial pressure (mmHg) | 80 (75-93) | | 81 (73-90) | 76 (70-87) | 77 (67-87) | 80 (72-95) | 80 (78-85) |
| Norepinephrine, n (%) | 46 (39) | | 30 (26) | 23 (28) | 13 (30) | 5 (23) | 3 (23) |
| Norepinephrine dosage (µg/kg/min) | 0.14 (0.05-0.29) | | 0.11 (0.05-0.32) | 0.13 (0.05-0.23) | 0.07 (0.04-0.18) | 0.12 (0.06-0.16) | 0.15 (0.14-0.26) |
|  |  | |  |  |  |  |  |
| Echocardiographic variables |  | |  |  |  |  |  |
|  |  | |  |  |  |  |  |
| LV ejection fraction (%) | 62 (55- 69) | | 65 (58-70) | 65 (60-72) | 67 (60-74) | 67 (61-72) | 68 (60-71) |
| Velocity-time integral of the LV outflow tract (cm) | 22 (18-24) | | 22 (19-26) | 21 (17-25) | 23 (20-27) | 23 (21-26) | 23 (21-26) |
| E/A ratio | 0.95 (0.80-1.17) | | 0.97 (0.79-1.13) | 0.87 (0.69-1.13) | 0.84 (0.72-1.06) | 1.00 (0.80-1.10) | 0.91 (0.75-1.29) |
| e’_septal_ (cm/s) | 8.9 (7.6-10.8) | | 8.7 (7.1-11.0) | 8.6 (7.1-11.4) | 8.5 (6.9-11.3) | 9.5 (8.0-11.7) | 8.9 (7.4-11.0) |
| e’_lateral_ (cm/s) | 9.6 (8.0-12.0) | | 11.1 (8.9-12.2) | 10.0 (8.0-12.0) | 10.0 (9.0-12.0) | 10.1 (8.0-12.0) | 9.6 (8.0-12.2) |
| E/e’_averaged_ | 8.15 (6.54-9.50) | | 7.60 (6.38-9.15) | 7.33 (6.18-9.29) | 7.56 (6.67-9.27) | 8.60 (7.10-10.00) | 7.61 (6.35-10.21) |
| TAPSE (mm) | 21 (18-25) | | 22 (19-25) | 22 (19-25) | 22 (20-27) | 22 (19-28) | 20 (18-23) |
| Systolic tricuspid annular velocity (cm/s) | 14 (12-17) | | 15 (13-18) | 17 (14-19) | 16 (13-20) | 17 (16-19) | 16 (14-19) |
| RV FAC (%) | 46 (40-54) | | 48 (42-54) | 48 (42-55) | 50 (44-60) | 50 (42-56) | 47 (41-53) |
| RV/LV end-diastolic areas ratio | 0.37 (0.17-0.57) | | 0.36 (0.18-0.54) | 0.40 (0.18-0.56) | 0.39 (0.21-0.51) | 0.44 (0.35-0.56) | 0.43 (0.38-0.52) |
| Systolic pulmonary arterial pressure (mmHg) | 29 (22-36) | | 30 (22-35) | 30 (25-38) | 29.1 (21.2-39.4) | 31 (25-39) | 38 (33-43) |
| Paradoxical septal motion, n (%) | 2 (2)  2) | | 1 (1) | 1 (1) | 0 (0) | 0 (0) | 0 (0) |
| TAPSE/SPAP (mm/mmHg)* | 0.74 (0.51-0.89) | | 0.74 (0.56-1.00) | 0.66 (0.49-0.88)  )) | 0.81 (0.65-1.07)  ) | 0.80 (0.62-0.97) | 0.54 (0.49-0.60)  ) |
| RV FAC/SPAP (%/mmHg)* | 1.60 (1.25-2.18) | | 1.66 (1.29-2.10)  ) | 1.55 (1.11-1.94)  ) | 1.71 (1.22-2.29)  ) | 1.63 (1.14-2.04)  ) | 1.24 (1.05-1.35)  ) |
|  |  | |  |  |  |  |  |
| *Variables are summarized as median (interquartile range) and numbers (percentages).*  ** SPAP measurement available in 62 patients.*  *ICU: intensive care unit; TTE: transthoracic echocardiography; LV : left ventricular; E: early peak velocity of transmitral flow with pulsed Doppler; A: atrial peak velocity of transmitral flow with pulsed Doppler; e’: early diastolic peak velocity of the mitral annulus with tissue Doppler imaging; TAPSE : tricuspid annular plane systolic excursion ; RV FAC : right ventricular fractional area change; SPAP: systolic pulmonary artery pressure.* | | | | | | | |

| **Table S4. Ventilatory settings, oxygenation and hemodynamic variables when pooling all TTE examinations during ICU stay according to the RV injury pattern.** | | | | | | |
| --- | --- | --- | --- | --- | --- | --- |
|  | **No RV injury**  **(n=267)** | **Isolated RV dilatation**  **(n=61)** | **RV dysfunction without RV dilatation**  **(n=50)** | **RV dysfunction with RV dilatation**  **(n=12)** | **Acute cor pulmonale (n=3)** | **p-value** |
| **Ventilatory settings at the time of TTE examination** |  |  |  |  |  |  |
|  |  |  |  |  |  |  |
| Standard oxygenation, n (%) | 6 (2) | 3 (5) | 0 (0) | 0 (0) | 0 (0) | <0.01 |
| High-flow nasal oxygen therapy, n (%) | 86 (32) | 14 (23) | 16 (32) | 0 (0) | 0 (0) | 0.08 |
| Non invasive ventilation, n (%) | 1 (1) | 2 (3) | 3 (6) | 0 (0) | 0 (0) | 0.03 |
| Intubation, n (%) | 174 (65) | 39 (64) | 31 (62) | 12 (100) | 3 (100) | 0.08 |
| Positive end-expiratory pressure (cmH20) | 12 (8-14) | 10 (8-12) | 12 (10-14) | 10 (10-12) | 20 (12-20) | <0.01 |
| Driving pressure (cmH2O) | 12 (9-14) | 13 (9-20) | 11 (9-13) | 15 (12-17) | 12 (10-14) | 0.08 |
| Compliance of the respiratory system (mL/cmH2O) | 33 (27-44) | 27 (16-38) | 36 (28-44) | 26 (23-33) | 41 (32-59) | 0.01 |
|  |  |  |  |  |  |  |
| **Oxygenation variables at the time of TTE examination** |  |  |  |  |  |  |
|  |  |  |  |  |  |  |
| PaO_2_/FiO_2_ | 161 (121-218) | 145 (105-193) | 133 (100-209) | 185 (149-272) | 115 (54-178) | 0.02 |
| PaCO_2_ (mmHg) | 38 (34-44) | 43 (37-51) | 37 (33-42) | 42 (38-47) | 46 (42-48) | 0.001 |
|  |  |  |  |  |  |  |
| **Hemodynamic variables at the time of TTE examination** |  |  |  |  |  |  |
|  |  |  |  |  |  |  |
| Heart rate (bpm) | 79 (66-97) | 78 (69-90) | 81 (70- 97) | 85 (61-104) | 121 (103-157) | 0.12 |
| Systolic arterial pressure (mmHg) | 122 (108-137) | 120 (112-128) | 123 (109-132) | 107 (99-128) | 101 (100-14) | 0.46 |
| Diastolic arterial pressure (mmHg) | 60 (53-66) | 56 (51-62) | 63 (56-71) | 61 (50-72) | 65 (50-82) | <0.01 |
| Mean arterial pressure (mmHg) | 80 (72-89) | 76 (71-82) | 81 (75-90) | 75 (68-94) | 66 (48-101) | 0.14 |
| Norepinephrine, n (%) | 71 (27) | 20 (33) | 20 (40) | 7 (58) | 2 (67) | 0.03 |
|  |  |  |  |  |  |  |
| *n=393 TTE examinations. Variables are summarized as median (interquartile range) and numbers (percentages).*  *ICU: intensive care unit; FiO_2_: inspired fraction of oxygen; PaO_2_ : partial arterial pressure of oxygen; PaCO_2_ : partial arterial pressure of carbon dioxide; RV: right ventricular; TTE: transthoracic echocardiography.* | | | | | | |

| Table S5. Number of patients with *de novo* RV injury during ICU stay. | | | | | | |
| --- | --- | --- | --- | --- | --- | --- |
|  | **ICU admission** | **Day-3** | **Day-7** | **Day-14** | **Day-21** | **Day-28** |
| Number of patients | 118 | 117 | 80 | 43 | 22 | 13 |
|  |  |  |  |  |  |  |
| Isolated RV dilatation, n (%) | 18 (15) | 2 (2) | 3 (4) | 2 (5) | 0 (0) | 0 (0) |
| RV dysfunction without RV dilatation, n (%) | 28 (24) | 3 (3) | 3 (4) | 2 (5) | 0 (0) | 0 (0) |
| RV dysfunction with RV dilatation, n (%) | 5 (4) | 2 (2) | 2 (3) | 1 (2) | 0 (0) | 0 (0) |
| Acute cor pulmonale, n (%) | 2 (2) | 0 (0) | 0 (0) | 0 (0) | 0 (0) | 0 (0) |
|  |  |  |  |  |  |  |
| *Variables are expressed as numbers.*  *ICU: intensive care unit; RV: right ventricular.* | | | | | | |

| **Table S6. Ventilatory settings, oxygenation and hemodynamic variables at TTE examination before and at the time of RV injury diagnosis in patients without RV injury on ICU admission.** | | | |
| --- | --- | --- | --- |
|  | **TTE before RV injury (n=20)** | **First TTE with RV injury**  **(n=20)** | **p-value** |
| **RV injury pattern** |  |  | . |
|  |  |  |  |
| No RV injury, n (%) | 20 (100) | 0 (0) | <0.01 |
| Isolated RV dilation, n (%) | 0 (0) | 8 (40) | 0.22 |
| RV dysfunction without RV dilation, n (%) | 0 (0) | 10 (50) | 0.32 |
| RV dysfunction with RV dilation, n (%) | 0 (0) | 2 (10) | 0.29 |
| Acute cor pulmonale, n (%) | 0 (0) | 0 (0) | - |
|  |  |  |  |
| **Ventilatory settings at the time of TTE examination** |  |  |  |
|  |  |  |  |
| Standard oxygenation, n (%) | 1 (5) | 0 (0) | 0.31 |
| High-flow nasal oxygen therapy, n (%) | 3 (15) | 3 (15) | 0.59 |
| Non invasive ventilation, n (%) | 0 (0) | 0 (0) | - |
| Intubation, n (%) | 16 (80) | 17 (85) | 0.62 |
| Positive end-expiratory pressure (cmH20) | 12 (12-14) | 10 (8 -14) | 0.10 |
| Driving pressure (cmH2O) | 11 (8-13) | 12 (8-19) | 0.48 |
| Compliance of the respiratory system (mL/cmH2O) | 35 (31-42) | 32 (24-44) | 0.58 |
|  |  |  |  |
| **Oxygenation variables at the time of TTE examination** |  |  |  |
|  |  |  |  |
| PaO_2_/FiO_2_ | 183 (114-228) | 158 (134-235) | 0.49 |
| PaCO_2_ (mmHg) | 42 (36-47) | 42 (35-50) | 0.40 |
|  |  |  |  |
| **Hemodynamic variables at the time of TTE examination** |  |  |  |
|  | 30 (67) | 57 (78) | 0.17 |
| Heart rate (bpm) | 78 (70-97) | 79 (73-90) | 0.61 |
| Systolic arterial pressure (mmHg) | 121 (109-129) | 119 (108-131) | 0.81 |
| Diastolic arterial pressure (mmHg) | 60 (56-64) | 60 (53-64) | 0.70 |
| Mean arterial pressure (mmHg) | 80 (75-85) | 77 (69-88) | 0.23 |
| Norepinephrine, n (%) | 6 (30) | 7 (35) | 0.19 |
|  |  |  |  |
| *n=20 patients. Variables are expressed as median (interquartile) or numbers (percentages).*  *FiO_2_: inspired fraction of oxygen; ICU: intensive care unit; PaO_2_ : partial arterial pressure of oxygen; PaCO_2_ : partial arterial pressure of carbon dioxide; RV: right ventricular; TTE: transthoracic echocardiography.* | | | |

| Table S7. Risk factors for Day-28 mortality. | | | | | | | | | |
| --- | --- | --- | --- | --- | --- | --- | --- | --- | --- |
|  |  |  | **Univariate analysis** | | | **Multivariate analysis** | | | |
|  | ***Alive (n=95)*** | ***Dead (n=23)*** | ***HR*** | ***95% CI*** | ***p-value*** | ***aHR*** | ***95% CI*** | ***p-value*** |  |
| Clinical characteristics |  |  |  |  |  |  |  |  |  |
|  |  |  |  |  |  |  |  |  |  |
| Age > 65 years | 43 (45) | 15 (65) | 2.37 | (1.43-3.92) | <0.01 | 2.26 | (1.29-3.95) | <0.01 |  |
| Male | 67 (70) | 17 (74) | 1.32 | (0.76-2.27) | 0.32 |  |  |  |  |
| SOFA score on ICU admission > 7 | 46 (48) | 12 (52) | 0.65 | (0.39-1.07) | 0.09 |  |  |  |  |
| Obesity (BMI > 30 kg/m²) | 32 (34) | 8 (35) | 1.07 | (0.64-1.78) | 0.81 |  |  |  |  |
| Arterial hypertension | 40 (42) | 18 (78) | 4.32 | (2.40-7.79) | <0.01 |  |  |  |  |
| Diabetes mellitus | 24 (25) | 11 (48) | 2.02 | (1.26-3.23) | <0.01 |  |  |  |  |
| Dyslipidemia | 20 (21) | 11 (48) | 2.65 | (1.64-4.30) | <0.01 |  |  |  |  |
| Cardiovascular chronic disease* | 9 (10) | 7 (30) | 3.30 | (1.98-5.49) | <.01 | 2.78 | (1.60-4.82) | <0.01 |  |
| Immunosupression | 14 (15) | 10 (43) | 3.66 | (2.27-5.89) | <0.01 | 3.53 | (2.14-5.83) | <0.01 |  |
| Chronic respiratory disease | 2 (2) | 3 (13) | 2.42 | (1.08-5.40) | 0.03 |  |  |  |  |
| Chronic kidney disease | 12 (13) | 8 (35) | 3.47 | (2.14-5.62) | <0.01 |  |  |  |  |
| Renin-Angiotensin System Blockers | 31 (33) | 15 (65) | 3.26 | (2.00-5.31) | <0.01 |  |  |  |  |
| Time from onset of symptoms to ICU admission > 9 days | 40 (42) | 8 (35) | 0.63 | (0.38-1.05) | 0.07 |  |  |  |  |
|  |  |  |  |  |  |  |  |  |  |
| Biological variables on ICU admission |  |  |  |  |  |  |  |  |  |
|  |  |  |  |  |  |  |  |  |  |
| Leukocytes > 10 G/L | 34 (36) | 12 (52) | 1.46 | (0.91-2.33) | 0.12 |  |  |  |  |
| Neutrophils > 8 G/L | 35 (37) | 11 (48) | 1.42 | (0.89-2.27) | 0.14 |  |  |  |  |
| Lymphocytes < 0.8 G/L | 59 (62) | 17 (74) | 1.80 | (1.05-3.11) | 0.03 |  |  |  |  |
| Fibrinogen > 8 g/dL | 17 (18) | 7 (30) | 2.06 | (1.26-3.38) | <0.01 |  |  |  |  |
| D-Dimers > 1000 µg/L | 48 (50) | 19 (83) | 3.42 | (1.87-6.27) | <0.01 |  |  |  |  |
| Protein C reactive > 130 mg/L | 47 (49) | 12 (52) | 1.16 | (0.72-1.85) | 0.54 |  |  |  |  |
| Procalcitonin > 0.5 ng/L | 34 (36) | 9 (39) | 0.89 | (0.55-1.45) | 0.64 |  |  |  |  |
| Ferritin > 1000 ng/mL | 40 (42) | 10 (43) | 0.85 | (0.52-1.39) | 0.51 |  |  |  |  |
| Interleukin-6 > 66 pg/mL | 41 (43) | 12 (52) | 1.13 | (0.71-1.81) | 0.60 |  |  |  |  |
|  |  |  |  |  |  |  |  |  |  |
| Variables at the time of TTE examination |  |  |  |  |  |  |  |  |  |
|  |  |  |  |  |  |  |  |  |  |
| Intubation | 65 (68) | 20 (87) | 1.30 | (0.74-2.28) | 0.36 |  |  |  |  |
| Positive end-expiratory pressure > 12 cmH2O | 65 (68) | 20 (87) | 1.20 | (0.69-2.09) | 0.51 |  |  |  |  |
| Driving pressure > 15 cmH2O | 22 (23) | 13 (56) | 3.38 | (1.95-5.86) | <0.01 |  |  |  |  |
| PaO_2_/FiO_2_ < 100 | 41 (43) | 13 (56) | 1.36 | (0.74-2.51) | 0.32 |  |  |  |  |
| PaCO_2_ > 45 mmHg | 32 (34) | 13 (56) | 1.68 | (0.99-2.85) | 0.05 | 2.21 | (1.22-4.02) | <0.01 |  |
| Norepinephrine | 51 (54) | 17 (74) | 1.84 | (1.12-3.01) | 0.02 |  |  |  |  |
| Norepinephrine dosage > 0.25 µg/kg/min | 15 (16) | 8 (35) | 1.57 | (0.74-3.32) | 0.24 |  |  |  |  |
|  |  |  |  |  |  |  |  |  |  |
| RV injury pattern |  |  |  |  |  |  |  |  |  |
|  |  |  |  |  |  |  |  |  |  |
| No RV injury | 42 (44) | 3 (13) | 1.00 | - | 0.06 | 1.00 | - | 0.11 |  |
| Isolated RV dilation | 17 (18) | 8 (35) | 1.49 | (0.78-2.84) | 0.35 | 1.55 | (0.74-3.26) | 0.25 |  |
| RV dysfunction without RV dilation | 30 (32) | 6 (26) | 1.76 | (0.87-3.55) | 0.15 | 1.61 | (0.70-3.72) | 0.24 |  |
| RV dysfunction with RV dilation or acute cor pulmonale** | 6 (6) | 6 (26) | 3.48 | (1.54-7.86) | 0.01 | 3.18 | (1.16-8.74) | 0.03 |  |
|  |  |  |  |  |  |  |  |  |  |
| *n=118 patients. Variables are expressed as numbers (percentages).*  **Cardiovascular chronic disease = coronary artery disease + stroke + chronic heart failure.*  *** RV dysfunction with RV dilation and acute cor pulmonale were combined because of the low number of patients with acute cor pulmonale (n=2).*  *All variables at the time of TTE examination and RV injury pattern were considered as time-dependent covariates.*  *aHR: adjusted hazard ratio; CI: confidence interval; ECMO: extracorporeal membrane oxygenation; FiO_2_: inspired fraction of oxygen; ICU: intensive care unit; PaO_2_: partial arterial pressure of oxygen; PaCO_2_: partial arterial pressure of carbon dioxide; RV: right ventricular; SOFA: sepsis-related organ failure assessment; TTE: transthoracic echocardiography.* | | | | | | | | |  |

| Table S8. Mixed effect logistic regression to assess the impact of RV injury on the Day-28 mortality rate. | | | | | | |
| --- | --- | --- | --- | --- | --- | --- |
|  | **Univariate analysis** | | | **Multivariate analysis** | | |
|  | ***OR*** | ***95% CI*** | ***p-value*** | ***aOR*** | ***95% CI*** | ***p-value*** |
| Variables |  |  |  |  |  |  |
|  |  |  |  |  |  |  |
| Age > 65 years |  |  |  | 2.46 | (0.53-11.50) | 0.25 |
| Cardiovascular chronic disease* |  |  |  | 9.45 | (1.43-62.32) | 0.02 |
| Immunosupression |  |  |  | 9.62 | (1.85-50.16) | 0.01 |
|  |  |  |  |  |  |  |
| Variables at the time of TTE examination |  |  |  |  |  |  |
|  |  |  |  |  |  |  |
| PaCO_2_ > 45 mmHg |  |  |  | 2.48 | (0.65-9.44) | 0.18 |
|  |  |  |  |  |  |  |
| RV injury pattern |  |  |  |  |  |  |
|  |  |  |  |  |  |  |
| No RV injury | 1.00 | - | 0.41 | 1.00 | - | 0.58 |
| Isolated RV dilation | 2.02 | (0.49-8.24) | 0.33 | 1.28 | (0.26-6.24) | 0.76 |
| RV dysfunction without RV dilation | 1.40 | (0.34-5.75) | 0.64 | 1.60 | (0.33-7.72) | 0.56 |
| RV dysfunction with RV dilation or acute cor pulmonale** | 5.41 | (0.60-49.10) | 0.13 | 5.45 | (0.45-66.10) | 0.18 |
|  |  |  |  |  |  |  |
| *n=118 patients. The variables introduced into the model were selected on the basis of the results of the time-dependent Cox model (Table S7).*  **Cardiovascular chronic disease = coronary artery disease + stroke + chronic heart failure.*  *** RV dysfunction with RV dilation and acute cor pulmonale were combined because of the low number of patients with acute cor pulmonale (n=2).*  *aOR: adjusted odds ratio; CI: confidence interval; PaCO_2_: partial arterial pressure of carbon dioxide; RV: right ventricular; TTE: transthoracic echocardiography.* | | | | | | |
