# Supplementary figures and images for: Right ventricular injury in critically ill patients with COVID-19: a descriptive study with standardized echocardiographic follow-up
Source: Ann Intensive Care. 2024 Jan 23;14:14. doi: 10.1186/s13613-024-01248-8 (PMC10805901; doi:10.1186/s13613-024-01248-8)

## Slide 1
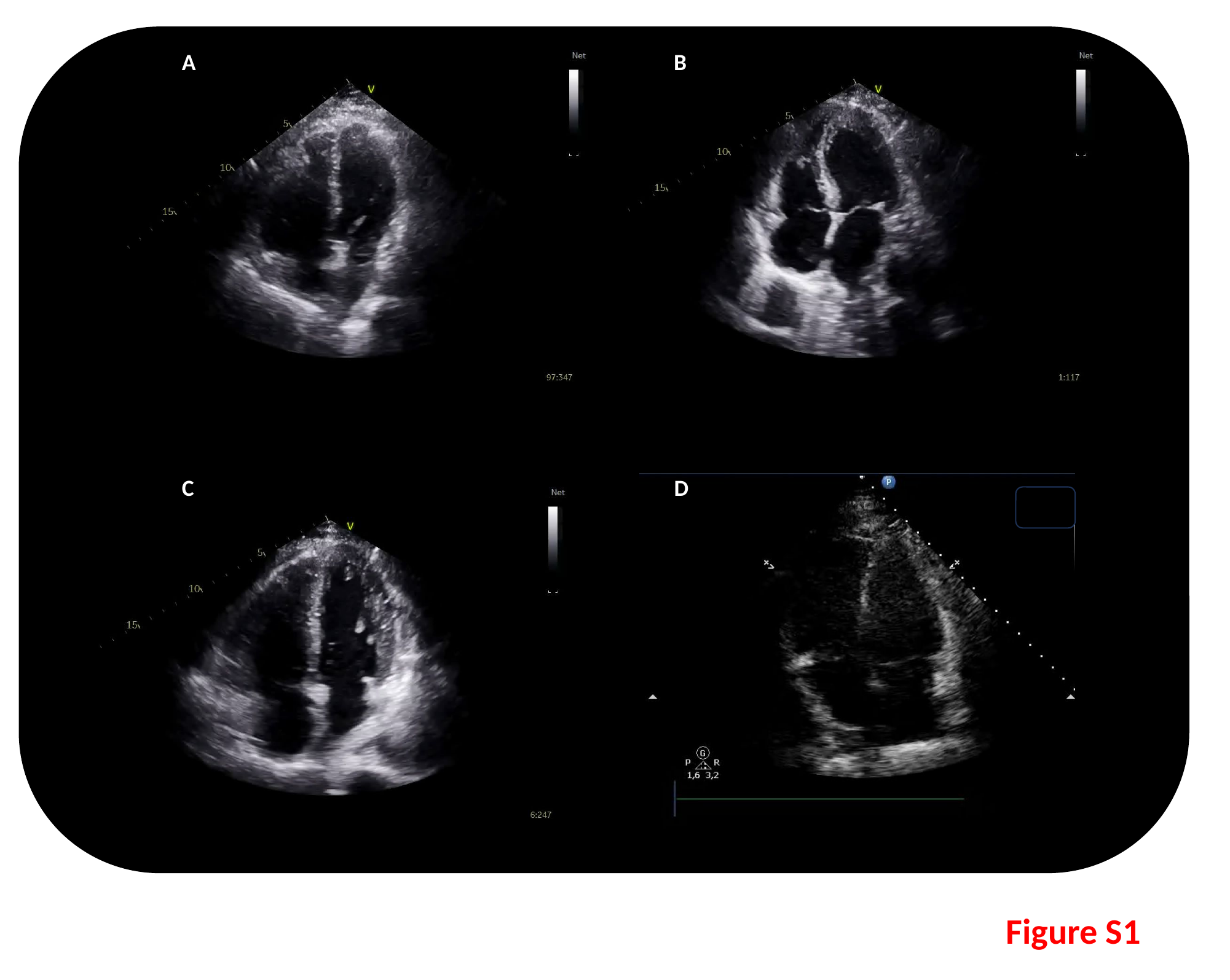

A
B
C
D
Figure S1

Supplement: Supplementary file 2 — Additional file 2: Figure S1. Echocardiographic loops illustrating the four different right ventricular (RV) injury patterns. Panel A: isolated RV dilation (apical 4-chamber view). Panel B: RV dysfunction without RV dilation (apical 4-chamber view). Panel C: RV dysfunction with RV dilation (apical 4-chamber view). Panel D: acute cor pulmonale with paradoxical septal motion (apical 4-chamber view). [file 13613_2024_1248_MOESM2_ESM.pptx]
